# Early activation and recruitment of invariant natural killer T cells during liver ischemia-reperfusion: the major role of the alarmin interleukin-33

**DOI:** 10.3389/fimmu.2023.1099529

**Published:** 2023-05-09

**Authors:** Aurélie Robin, Claire Mackowiak, Romain Bost, Fanny Dujardin, Alice Barbarin, Antoine Thierry, Thierry Hauet, Luc Pellerin, Jean-Marc Gombert, Ephrem Salamé, André Herbelin, Louise Barbier

**Affiliations:** ^1^ Centre Hospitalier Universitaire de Poitiers, Institut National de la Santé Et de la Recherche Médicale, Ischemie Reperfusion Métabolisme et Inflammation Stérile en Transplantation, Université de Poitiers, Poitiers, France; ^2^ Institut National de la Santé Et de la Recherche Médicale (INSERM), Ischemie Reperfusion Métabolisme et Inflammation Stérile en Transplantation (IRMETIST), Université de Poitiers, Poitiers, France; ^3^ Centre Hospitalier Universitaire (CHU) Trousseau, Pathology, Tours, France; ^4^ Université de Poitiers, Institut National de la Santé Et de la Recherche Médicale (INSERM), Ischemie Reperfusion Métabolisme et Inflammation Stérile en Transplantation (IRMETIST), Centre Hospitalier Universitaire (CHU) de Poitiers, Nephrology, Poitiers, France; ^5^ Université de Poitiers, Institut National de la Santé Et de la Recherche Médicale (INSERM), Ischemie Reperfusion Métabolisme et Inflammation Stérile en Transplantation (IRMETIST), Centre Hospitalier Universitaire (CHU) de Poitiers, Biochemistry, Poitiers, France; ^6^ Université de Poitiers, Institut National de la Santé Et de la Recherche Médicale (INSERM), Ischemie Reperfusion Métabolisme et Inflammation Stérile en Transplantation (IRMETIST), Centre Hospitalier Universitaire (CHU) de Poitiers, Immunology, Poitiers, France; ^7^ Université de Tours, Centre Hospitalier Universitaire (CHU) Trousseau, Digestive Surgery and Liver Transplantation, Institut National de la Santé Et de la Recherche Médicale (INSERM), Ischemie Reperfusion Métabolisme et Inflammation Stérile en Transplantation (IRMETIST), Tours, France

**Keywords:** innate like T cell, iNKT cell, interleukin-33, sterile Inflammation, alarmin, liver transplantation, ischemia - reperfusion

## Abstract

Over the past thirty years, the complexity of the αβ-T cell compartment has been enriched by the identification of innate-like T cells (ITCs), which are composed mainly of invariant natural killer T (iNKT) cells and mucosal-associated invariant T (MAIT) cells. Based on animal studies using ischemia-reperfusion (IR) models, a key role has been attributed to iNKT cells in close connection with the alarmin/cytokine interleukin (IL)-33, as early sensors of cell-stress in the initiation of acute sterile inflammation. Here we have investigated whether the new concept of a biological axis of circulating iNKT cells and IL-33 applies to humans, and may be extended to other ITC subsets, namely MAIT and γδ-T cells, in the acute sterile inflammation sequence occurring during liver transplant (LT). From a prospective biological collection of recipients, we reported that LT was accompanied by an early and preferential activation of iNKT cells, as attested by almost 40% of cells having acquired the expression of CD69 at the end of LT (i.e. 1-3 hours after portal reperfusion), as opposed to only 3-4% of conventional T cells. Early activation of iNKT cells was positively correlated with the systemic release of the alarmin IL-33 at graft reperfusion. Moreover, in a mouse model of hepatic IR, iNKT cells were activated in the periphery (spleen), and recruited in the liver in WT mice, as early as the first hour after reperfusion, whereas this phenomenon was virtually missing in IL-33-deficient mice. Although to a lesser degree than iNKT cells, MAIT and γδ-T cells also seemed targeted during LT, as attested by 30% and 10% of them acquiring CD69 expression, respectively. Like iNKT cells, and in clear contrast to γδ-T cells, activation of MAIT cells during LT was closely associated with both release of IL-33 immediately after graft reperfusion and severity of liver dysfunction occurring during the first three post-operative days. All in all, this study identifies iNKT and MAIT cells in connection with IL-33 as new key cellular factors and mechanisms of acute sterile inflammation in humans. Further investigations are required to confirm the implication of MAIT and iNKT cell subsets, and to precisely assess their functions, in the clinical course of sterile inflammation accompanying LT.

## Introduction

Besides classic adaptive CD4+ and CD8+ T cells, the T cell compartment comprises several lineages of cells endowed with both innate and adaptive properties that are referred to as unconventional or innate-like invariant T cells (ITCs) (for reviews (1–3):). This class of T cells includes three main lineages, i.e., invariant natural killer T (iNKT) cells, mucosa-associated invariant T (MAIT) cells, and γδ-T cells. These cells recognize non-peptide antigens, are not restricted to classical major histocompatibility complex (MHC) and have emerged as key players in tissue homeostasis and protection from infection. As immune system components, ITCs are also expected to rapidly and efficiently respond to cell-stress and represent obvious candidates to participate in endogenous pathways of inflammation. For instance, non-classical MHC cluster differentiation (CD)1d-restricted presentation of self-antigens to iNKT cells is induced by endogenous stress and may be activated by proinflammatory cytokines (4, 5). Given their versatile functions, in addition to contributing to tissue inflammation and damage, ITCs can participate in the process of the resolution of inflammation, including tissue repair and regeneration (6, 7).

Based on numerous mouse models, iNKT cells are recognized as the archetypal ITCs, which are capable of being early sensors and managers in the development and resolution of acute sterile inflammation (for review: (8)), a condition that occurs in the absence of infection and underlies medical afflictions such as mechanical trauma, chemical and environmental insults, and organ ischemia-reperfusion (IR) injury (IRI) (for reviews: (9–11)). Indeed, activation of iNKT cells has been documented as a general mechanism for the initiation of IRI in solid organs (kidney, liver, lung, brain and heart) ( (12–18); for review: (8)). Moreover, exploration of the modes of activation and recruitment of iNKT cells in sterile inflammation induced by IRI in mice suggests the involvement of T cell receptor (TCR)/non-classical MHC CD1d molecule interactions in iNKT cell activation, even though further investigations are needed to characterize the effective endogenous lipids presented by CD1d (13, 15, 17, 19–21). Another specific feature of ITCs is their ability to be activated by cytokine-driven signals independently of TCR-engagement and CD1d molecule recognition. In particular, the pro-inflammatory cytokines interleukin (IL)-12 and IL-18 can activate iNKT cells to produce IFN-γ, a response that is amplified upon TCR-engagement (22). Accordingly, in a model of sterile liver injury, the authors documented a biphasic mechanism of iNKT cell activation through self-antigen presentation and IL-12/IL-18-driven signals (7).

More recently, the alarmin/cytokine IL-33 has emerged as a new general mechanism driving activation and recruitment of iNKT cells in the early phase of sterile inflammation (for review:(8)). IL-33 belongs to the IL-1 superfamily ((23); for review: (24)), is constitutively expressed in the nucleus of endothelial cells and epithelial cells and is immediately released after tissue damage ( (12, 25–31); for reviews: (32, 33)). IL-33 has aroused interest due to its singular action as an alarmin during infectious, allergic responses and acute tissue injury (8, 24, 29, 34, 35), and as a cytokine, contributing to the resolutive/repair phase of sterile inflammation (30, 31, 36, 37). We recently provided evidence that IL-33 and iNKT cells interact directly to promote kidney inflammation in an experimental model of renal IRI (12). Indeed, iNKT cells, like innate cells (natural killer cells, neutrophils), constitutively express the IL-33 receptor-specific ST2 chain (38, 39). As a result, recruitment of iNKT cells is rapidly driven by endogenous released IL-33, which induces their IFN-γ/IL-17A production in response to IR (12).

Even though the contribution of iNKT cells to induction of sterile inflammation in experimental models is well-established, the place of this ITC subset in acute conditions resulting from sterile inflammation in humans remains poorly documented. Considering our recent demonstration that endogenous IL-33 acts as an alarmin in liver IR and is associated with injury after human liver transplantation (LT) (40), we decided to investigate whether the new concept of a biological axis of iNKT cells and IL-33, involved in alerting the immune cells during sterile inflammation, applies to human LT.

To this end, we analyzed the influence of the LT on the activation status of blood iNKT cells and its possible association with early IL-33 release. To conclusively demonstrate that IL-33 is the causal effect, we compared iNKT cell activation in wild-type (WT) and IL-33-deficient mice in a model of hepatic IRI. We extended our analysis in LT patients to the other main ITC compartments, namely MAIT and γδ-T cells, relative to the adaptive conventional T cell compartments.

## Material and methods

### LT patients and healthy subjects

Twenty-one patients from a prospective biological collection of adult LT recipients from the Transplant Unit of the University Hospital of Tours between July 1, 2017, and June 2, 2019, were included. These patients represent a sub-group of patients described in a previous report (40) where intra-operative and post-operative management was described. Recipients, donor characteristics, and outcomes of LT are detailed in **Supplementary Table 1**.

Early allograft dysfunction was assessed by the model of early allograft function (MEAF) score defined by Pareja et al. in 2015 (41) (mathematical model based on bilirubinemia levels, International Normalized Ratio and ALT during the first three post-operative days, giving a continuous score ranging from 0 to 10).

Peripheral blood mononuclear cells (PBMC) from eleven healthy subjects (HS) (mean age 47 ± 15 years) without liver disease obtained from the French Blood Institute (Etablissement Français du Sang, Lyon, France) were used as controls. This study was approved by a regional ethics committee (comité consultatif de protection des personnes dans la recherche biomédicale Tours-Région Centre-Ouest 1 under registration number DC-2016-2651) and by the French regulatory agency (Agence de la Biomédecine, the national authority for organ procurement and transplantation in France, under registration number PFS16-005). Written informed consents were obtained for each patient and healthy donor according to the Declaration of Helsinki. All data were collected anonymously in a prospectively maintained database declared to the French Data Protection Authority. No potentially identifiable human images or data are presented in this study.

### PBMC and serum collections from LT patients

PBMC were isolated by Ficoll density gradient from peripheral blood of HS (n=11) and patients prior to LT (D0: usually 2-6 hours prior to transplantation; n=17), and at the end of the procedure during skin closure (EoT; n=16).

Serum was isolated from peripheral blood of healthy subjects (n=11) and LT patients at D0 (n=21), at graft reperfusion (GR: just after unclamping of the caval and portal anastomoses, usually around 3-4 hours after the beginning of the procedure; n=21), and at EoT (n=21). PBMC and serum were stored frozen in liquid nitrogen and at -80°C, respectively, at the Centre de Ressources Biologiques Santé of Tours (BB-0033-00013).

### Enzyme-linked immunosorbent assay

Serum IL-33, IL-12 and IL-18 (R&D systems) were determined using ELISA kits according to the manufacturer’s instructions.

### Mice

WT C57BL/6 mice were purchased from Janvier Labs (Le Genest-Saint-Isle, France). Jα18KO C57BL/6 mice (lacking iNKT cells), hereafter referred to as iNKT cell-deficient mice, were provided by Taniguchi and colleagues (42). Mice with a Lac-z gene-trap (Gt) reporter (IL-33^Gt/Gt^) onto a C57BL/6 background, hereafter referred to as IL-33-deficient mice, were generated as described by Pichery et al. (43). Ten-to-twelve-week-old male mice weighing between 22 and 30 g were used in all experiments.

### Experimental model of warm IRI

All animal experimental procedures and housing were carried out in accordance with the European Communities Council Directive (2010/63/EU) and complied with the three Rs principle (Replace, Reduce, Refine). The animal study was reviewed and approved by a regional Ethics Committee (COMETHEA Poitou-Charentes, authorization n°2016110211568800).

The entire procedure of warm IR was adapted from the technique described by Abe et al. (44). Briefly, thirty minutes before general anesthesia, mice received analgesia with subcutaneous injection of buprenorphine (0.05 mg/kg). Mice were then anaesthetized with intraperitoneal injection of ketamine (80 mg/kg) and xylazine (10 mg/kg). Mice then received continuous anesthesia with gaseous administration of isoflurane (2% then 1.5% for maintenance). After disinfection of the abdomen, laparotomy with right subcostal and upper midline incision was performed. Median and left lateral lobes were lift up to expose their hepatic pedicle just above the branching to the right lateral lobe and an atraumatic clamp was placed. This technique allows partial ischemia of 70% of the liver (median and left lateral lobes), while right lateral and caudate, quadrate lobes are still perfused. Saline serum was carefully added to the abdominal cavity for hydration. Clamp was applied for 70 minutes under gaseous isoflurane anesthesia. The clamp was then removed, and the abdominal wall and skin were totally closed before isoflurane cessation. Animals were culled at various time points after reperfusion by cervical dislocation.

In sham-operated animals, the surgical procedure was identical, but without clamping of the hepatic pedicle.

### Mouse plasma collection for ALT activity assay

Blood samples were obtained by retro-orbital bleed under gaseous isoflurane (5%) and collected in tubes containing heparin. After centrifugation (10 minutes, 2000g), plasma samples were stored at -20°C prior to ALT measurement with a Cobas C701 automatic analyzer (Roche Diagnostic).

### Histology of mouse livers

The portal vein was cut immediately after euthanasia and the inferior vena cava was injected with phosphate-buffered saline to perfuse the liver. Liver samples were preserved in formalin before paraffin embedding. Samples were cut at 3.5μm and subjected to hematoxylin phloxine saffron staining. To quantify tissue lesions of IRI, we used a reading grid from the literature that we adapted, as previously detailed (43). The total number of points was referred to as the liver injury score.

### Mouse cell isolation

Dissociation of liver and spleen was carried out at 37°C during 30 minutes and at room temperature during 56 seconds, respectively, using a gentleMACS™ dissociator (Miltenyi Biotec), according to the manufacturer’s instructions. Note that dissociation of liver was performed with the Liver Dissociation kit (Miltenyi Biotec) in gentleMACS™ C tubes (Miltenyi Biotec). Spleen and liver samples were then homogenized through a 70-μm cell-strainer and washed. Parenchymal cells were removed by centrifugation and red blood cells were lysed in ammonium chloride buffer before resuspension in DMEM cell culture media and RPMI 1640 for liver and spleen samples, respectively. Cell counting was performed in trypan blue. Cell numbers were determined with a hematocytometer, re-suspended at 1.10^6^/mL in phosphate-buffered saline, and expressed per mg tissue.

### Flow cytometry analysis

Briefly, cells were stained by viability dye, and then incubated with the appropriate antibody/reagent mix prior to fixation, as previously reported (12, 45). A detailed list of antibodies and reagents used for staining is provided in **Supplementary Table 2**. Fluorescence minus one control or unstained cells served as negative staining control samples. Samples were acquired on a spectral cytometer AURORA using SpectroFlo^®^ software for unmixing (Cytek) and on a BD FACS Verse™ cytometer using the BD FACsSuite software (BD Biosciences) for human and mouse samples, respectively. Data were analyzed using FlowJo v10 (BD Biosciences). Because of the very low relative proportion of iNKT cells among human PBMC (that can be less than 0.01% of total CD3(+) cells), a minimum of 50,000 CD3(+) cells were analyzed for all human samples. For qualitative evaluation (CD69 and CD4 expression), iNKT cells could be counted (mean ± SEM and range/sample) in all HS (387 ± 148 [30-4221]), and in 8 (out of 17) (127 ± 51 [13-399]) and 9 (out of 16) (40 ± 17 [10-2982]) patients before and at the end of LT, respectively.

Gating strategies are detailed in **Supplementary Figure 1** and **Supplementary Figure 11** for human and mouse samples, respectively. Heatmap analysis presented in **Supplementary Figure 4** was performed by OMIQ^®^ software.

### Statistical analysis

Statistical analysis was performed using Excel^®^ for Mac Os (Microsoft^®^ Corporation) and Graph Pad Prism software v7 (GraphPad, La Jolla, Inc.). Quantitative data are presented as percentages and absolute numbers. Qualitative data are presented as mean (± standard error of the mean (SEM)). Non-parametric tests were used because of the small number of samples per group. Experimental and control groups were compared with the unpaired (independent) two-tailed Mann-Whitney U test. Spearman tests were performed for correlation analysis. Results were considered to be statistically significant when *p<*0.05.

## Results

### Overall decrease in the relative proportion of circulating ITC subsets in patients awaiting LT

**Figure 1:**
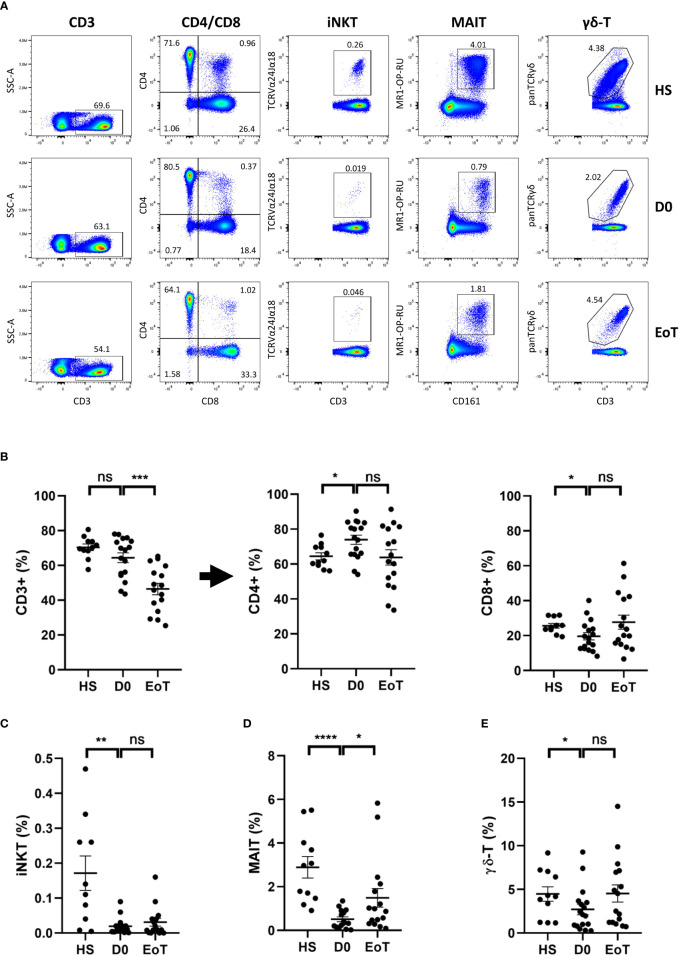
Effect of LT on relative proportion of blood conventional and ITC subsets. Flow cytometry analysis (representative plots are depicted in **(A)**) showing frequencies of total CD3(+) cells (among total mononuclear live cells), conventional CD4 T cells, referred as CD4+ cells, and conventional CD8 T cells, referred as CD8+ cells (among total CD3(+) cells) **(B)**, iNKT cells **(C)**, MAIT cells **(D)** and γδ-T cells **(E)** (among total CD3(+) cells) in peripheral blood of healthy subjects (HS; n=11), patients awaiting LT (D0; n=17) and after LT (EoT; n=16). Of note the decrease frequency of total T lymphocytes (CD3(+)) among PBMC between the beginning and the end of LT (64.4 ± 1.9% (n=17) *vs.* 46.4 ± 3.3% (n=16), respectively) is fully explained by an increase of its CD3(-) counterpart (53.6 ± 3.3% (n=16) at EoT *vs.* 35.6 ± 2.8% (n=17) at D0). For detailed gating strategy, see **Supplementary Figure 1**. Data are expressed as means ± SEM. Unpaired (independent) two-tailed Mann-Whitney U test. *p<0.05, **p<0.01, ***p<0.001, ****p<0.0001, ns, not significant. Each symbol represents one HS or a LT patient.

First, we analyzed the relative proportion of conventional lymphocytes and ITCs (for gating strategy, see **Supplementary Figure 1**) in patients awaiting LT (D0) and in healthy subjects (HS) (for representative plots, see **Figure 1A**). The frequency of total T lymphocytes (defined as CD3(+) cells) among PBMC did not differ between LT patients and HS (**Figure 1B**, left panel). In LT patients, we observed decreased frequency of the conventional CD8 T cell subset, as compared to HS (**Figure 1B**, right panel), which was accompanied by an increase of its CD4 counterpart (**Figure 1B**, middle panel). All in all, a dramatic decrease in frequency of all ITC components was evidenced among total T lymphocytes in LT patients as compared to HS (**Figures 1C-E**). Indeed, substantially lower frequency (mean ± SEM) of iNKT cells (0.02 ± 0.006 *vs.* 0.17 ± 0.02% in HS; *p<*0.01) (**Figure 1C**), MAIT cells (0.51 ± 0.11 *vs.* 2.89 ± 0.49% in HS; *p*<0.0001) (**Figure 1D**), and γδ-T cells (2.70 ± 0.61 *vs.* 4.47 ± 0.82% in HS; *p*<0.05) (**Figure 1E**) was observed.

### LT did not affect the relative proportion of ITC subsets, except for MAIT cells

Next, we investigated the impact of LT on frequencies of circulating conventional T lymphocytes and ITCs (**Figure 1**, for representative plots, see **Figure 1A**). As shown in **Figure 1B**, frequency of total T lymphocytes (CD3(+)) significantly decreased between the beginning (D0) and the end (EoT) of LT, a phenomenon explained by an increase of non-T lymphocytes (CD3(-)) among PBMC (for details, see the legend of **Figure 1**). Apart from CD4 T cells, for which a decreasing trend was observed, all T cell subsets showed an increased trend in terms of rate frequency, ranging from 1.4-fold-increase for conventional CD8 T-cells (**Figure 1B**), 1.6-fold-increase for iNKT cells (**Figure 1C**)), 1.6-fold-increase for γδ-T cells (**Figure 1E**) to a significant 2.9-fold-increase (*p*<0.05) for MAIT cells (**Figure 1D**).

### LT was associated with early and preferential activation of ITC subsets, especially iNKT cells

**Figure 2:**
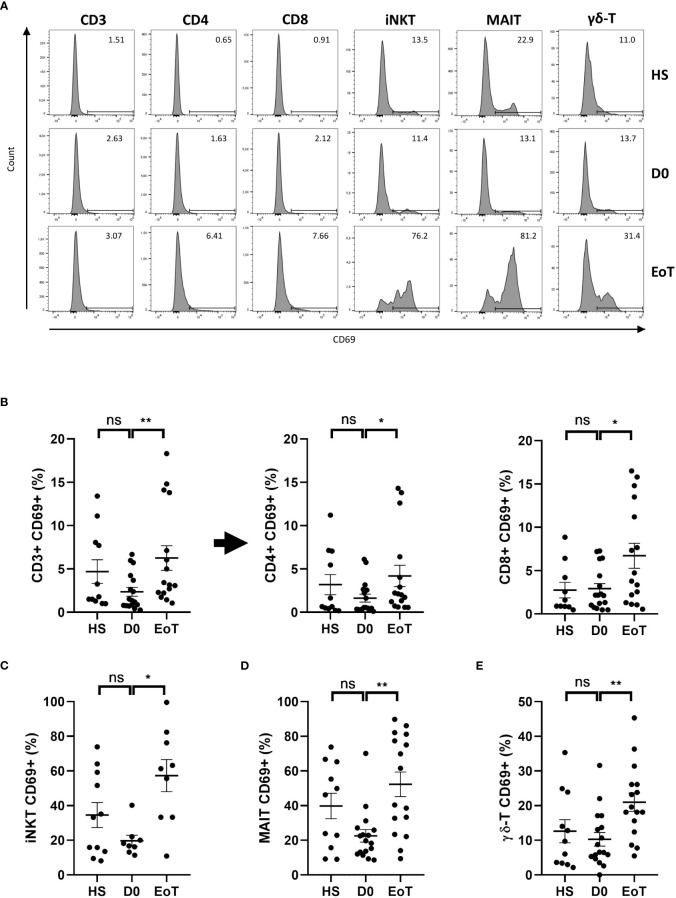
Effect of LT on relative proportion of blood CD69(+) cells among conventional and ITC subsets. Flow cytometry analysis (representative histograms are depicted in **(A)**) showing frequencies of CD69(+) cells among total CD3(+) cells, conventional CD4 T cells, referred as CD4+ cells, conventional CD8 T cells, referred as CD8+ cells **(B)**, iNKT cells **(C)**, MAIT cells **(D)**, and γδ-LT cells **(E)** in peripheral blood of healthy subjects (HS; n=11) patients before (D0; n=8-17) and at the end of LT (EoT; n=9-16). Of note, iNKT cells could not be detected in 9 (out of 17) and 7 (out of 16) patients before and at the end of LT, respectively. For detailed gating strategy, see **Supplementary Figure 1**. Representative plots are shown in **Supplementary Figure 2**. Data are expressed as means ± SEM. Unpaired (independent) two-tailed Mann-Whitney U test. *p<0.05, **p<0.01, ns, not significant. Each symbol represents one HS or LT patient.

LT was accompanied by cell activation of all T cell subsets (**Figure 2**; for representative histograms and plots, see **Figure 2A** and **Supplementary Figure 2**, respectively), as attested by the rate of increase in cells expressing the early activation marker CD69, which ranged from a 2.9-fold-increase for iNKT cells (**Figure 2C**), a 2.3-fold-increase for MAIT cells (**Figure 2D**) and conventional T cells (**Figure 2B**) to a 2.0-fold-increase for γδ-T cells (**Figure 2E**). Moreover, by comparing the percentage of cells expressing CD69 between the end and the beginning of LT, we deduced that almost 40% of iNKT cells had become activated (57.3 ± 9.2% at EoT *vs.* 19.7 ± 3.2% at D0), instead of only 3-4% of conventional T cells (CD3 T cells: 6.2 ± 1.4% at EoT *vs.* 2.3 ± 0.5 at D0; CD4 T cells: 4.2 ± 1.2% at EoT *vs.* 1.6 ± 3.6 at D0; CD8 T cells: 6.7 ± 1.4 at EoT *vs.* 2.9 ± 0.6% at D0). To an intermediate place, 30% of MAIT cells (52.3 ± 7.0 at EoT *vs.* 22.5 ± 3.6 at D0) and 10% of γδ-T cells (21.0 ± 2.6% at EoT *vs.* 10.3 ± 1.95% at D0) had also become activated during LT. Of note, iNKT cell activation during LT involved both CD4(+) (37.3 ± 11.3% at EoT *vs.* 9.55 ± 3.9% at D0) and CD4(-) (78.6 ± 8.6% at EoT *vs.* 37.0 ± 9.9% at D0) contingents (**Supplementary Figure 3A**). The same conclusion was reached when comparing the CD8(+) (55.4 ± 7.0% at EoT *vs.* 23.5 ± 3.8% at D0) and CD8(-) (47.4 ± 4.8% at EoT *vs.* 22.9 ± 4.8% at D0) contingents of the MAIT subset (**Supplementary Figure 3B**).

### Activation of iNKT and MAIT cells at the end of LT was closely associated with release of the alarmin IL-33 immediately after graft reperfusion

At this stage, an important question was to identify the underlying mechanism involved in activation of ITC populations during LT. Although TCR-engagement could not be excluded, the fact that no modulation of the TCR signal transduction molecule CD3 expression level was found on iNKT, MAIT and γδ-T cells between the beginning and the end of LT even when comparing CD69(+) to CD69(-) fractions for each of the three ITC populations studied, does not support the hypothesis of activation-induced TCR internalization (**Supplementary Figure 4**; for representative plots, see **Supplementary Figure 5**).

**Figure 3:**
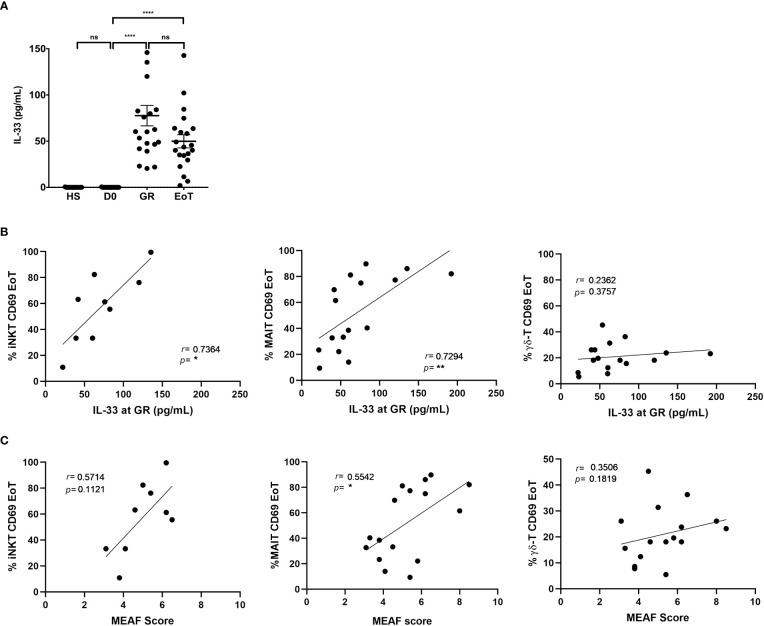
Analysis of the association between IL-33 release, ITC activation and liver injury in LT patients. **(A)** Serum levels of IL-33 (pg/mL) in healthy subjects (HS: n=11) and in patients prior to LT (D0: n=21), at graft reperfusion (GR: n=21), and at the end of LT (EoT: n=21). Horizontal bars represent means. Unpaired (independent) two-tailed Mann-Whitney U test. ****p<0.0001, ns, not significant. **(B, C)** Spearman’s rank correlation of CD69 expression (%) on blood ITC subsets (iNKT, MAIT, γδ-T cells) at the end of LT and serum levels of IL-33 at graft reperfusion (n=9-16) **(B)** or MEAF score (n=9-16) **(C)**. Of note, iNKT cells could not be detected in 7 out of 16 patients at the end of LT. Analysis was performed by Spearman test. Each symbol represents one LT patient.

Regarding innate activation by cytokines, as mentioned above, a major candidate is IL-33, which, on the one hand, targets innate components of the immune system, including ITCs such as iNKT cells (38, 39) and MAIT cells (46) and, on the other hand, has been documented to be released as an alarmin both in a mouse model of warm hepatic IRI and in the setting of human LT (40), a conclusion that we confirmed in the present study (**Figure 3A**).

Supporting the proposal that ITCs were activated early during LT in response to IL-33 release, we found a positive correlation between serum levels of IL-33 after graft reperfusion and expression of the early activation marker CD69 on blood iNKT cells (*r*=0.74; *p*<0.03; n=9) and MAIT cells (*r*=0.73; *p*<0.002; n=16) at the end of LT (**Figure 3B**; left and middle panel, respectively). In clear contrast, during LT, serum levels of IL-12 and IL-18, also known to activate ITCs, were neither increased (**Supplementary Figure 6A**), nor correlated with CD69 expression on any of the three ITC populations studied (**Supplementary Figure 6B**).

Note that no correlation was found with IL-33 when considering CD69(+) γδ-T cells (**Figure 3B**; right panel) and CD69(+) conventional T cells (**Supplementary Figure 7**). Moreover, and compatible with the hypothesis that IL-33 would be a common underlying factor contributing to iNKT and MAIT cell activation, we observed a positive correlation between the relative proportions of CD69(+) iNKT and CD69(+) MAIT cells at the end of LT. In contrast, CD69(+) γδ-T cells, which did not correlate with serum of IL-33, did not correlate with CD69(+) iNKT cells either (**Supplementary Figure 8**).

### Activation of iNKT and MAIT cells at the end of LT correlated with early graft dysfunction

Finally, we interrogated whether activation of ITCs induced by LT could be predictive of the level of early graft dysfunction, measured by the MEAF score (**Figure 3C**). Although no correlation was found for γδ-T cells (*r*=0.35; *p*=0.18; n=16), we observed that CD69 expression on MAIT cells (*r*=0.55; *p*<0.05; n=16) was positively correlated with the MEAF score. For iNKT cells, a non-significant trend (*p*=0.11) for a positive correlation was evidenced (*r*=0.57).

### The proof of concept of a deleterious IL-33/ITC axis applied to liver IR injury

In order to provide direct and definitive evidence of the causal link between IL-33 and activation of ITCs during liver IR, we decided to determine the impact of IL-33 on activation/recruitment of iNKT cells in a mouse model of warm hepatic ischemia. We chose to focus on iNKT cells, based on their presence in mouse at high frequencies both in periphery (spleen) and in liver (47), as well as their contribution to severity of liver IR, as attested by decreased ALT levels, and fewer neutrophil recruitment and histological lesions in iNKT cell-deficient (Jα18KO) mice, compared to WT mice (**Supplementary Figures 9**, **10**).

**Figure 4:**
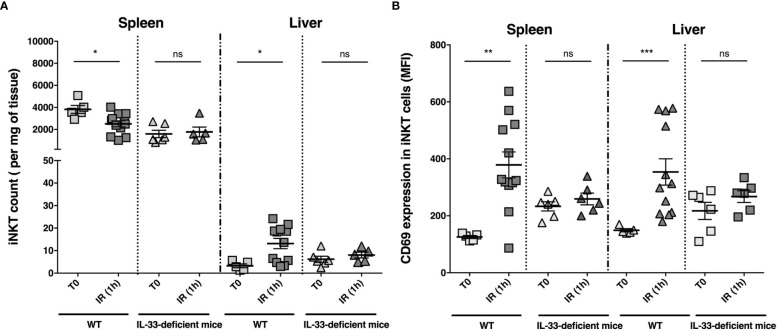
iNKT cell numbers and CD69 MFI on iNKT cells in spleen and liver before clamping and 1 hour after declamping of liver lobes: comparison between WT and IL-33-deficient mice. iNKT cell numbers (per milligram of tissue) **(A)** and CD69 expression (mean of fluorescence intensity (MFI)) on iNKT cells **(B)** were determined by flow cytometry in spleen and liver of WT and IL-33-deficient mice before liver clamping (T0) and after 1 hour after declamping of liver lobes (IR (1 h). Data are expressed as mean ± SEM. Of note, IL-33-dependent cell recruitment in the liver, although preferentially involving iNKT cells, tended to affect the entire CD3 T cell compartment (CD3(+) cell count/mg of tissue (mean ± SEM): 40 ± 17 at T0 (n=5) and 92 ± 17 (n=12) after 1 hour of reperfusion in WT mice; *p=*0.06 *vs.* 63 ± 23 (n=6) at T0 and 43 ± 4.6 (n=6) after 1 hour of reperfusion in IL-33-deficient mice; *p=*0.82). Unpaired (independent) two-tailed Mann-Whitney U test. *p<0.05, **p<0.01, ***p<0.001, ns, not significant. n=5-12 mice per group. Each symbol represents one mouse.

To test the hypothesis of an IL-33/iNKT cell axis, we then evaluated IL-33-deficient (IL-33^Gt/Gt^) mice (43) in our liver IR model. Number and activation level (mean fluorescence intensity (MFI) surface expression level of the early activation marker CD69) of iNKT cells were determined in the spleen and liver in WT and IL-33-deficient mice after 1 hour of reperfusion. Flow cytometry gating strategy to identify iNKT cells in mice is shown in **Supplementary Figure 11**. As depicted in **Figure 4**, the number of iNKT cells decreased in the spleen and increased in the liver of WT mice (**Figure 4A** and **Supplementary Figure 12**), while being activated (**Figure 4B** and **Supplementary Figure 13**). On the contrary, IL33-deficient mice showed neither recruitment (**Figure 4A**) nor activation of iNKT cells (**Figure 4B**) in the liver, demonstrating that recruitment and activation of iNKT cells after liver IR depends on IL-33.

## Discussion

This work provides initial evidence in humans of general and preferential activation of the circulating iNKT cell compartment in the acute sterile inflammation sequence occurring during LT.

Two lines of evidence support the hypothesis that early iNKT cell activation shown at the end of LT could be driven by the alarmin IL-33, which is released in circulation and reaches a peak immediately after graft reperfusion (40): **i)** activation of iNKT cells at the end of LT was closely associated with release of IL-33 immediately after graft reperfusion; **ii)** in a mouse model of liver IRI, early activation of iNKT cells depended on endogenous IL-33, as attested by its virtual loss when WT mice were replaced by IL-33-deficient mice.

In humans, although iNKT cells are rapidly activated in response to IL-33 alone (48), co-signals provided by the pro-inflammatory cytokines IL-12 and IL-18 or by TCR-engagement could also contribute to the amplitude of iNKT cell activation observed in patients at the end of LT. However, during LT, there was no correlation between serum levels of the two proinflammatory cytokines and activation of iNKT cells, suggesting that, unlike IL-33, IL-12 and IL-18 were not sufficient by themselves to significantly induce iNKT cell activation, even though we cannot exclude the involvement of IL-12 as an effective coagent of IL-33, due to its high baseline pre-LT levels. Furthermore, no TCR internalization on iNKT cells could be detected, which does not suggest involvement of a TCR ligand.

By providing the *princeps* observation that iNKT cells could be activated in response to IL-33 released as an alarmin during LT, our study extends to humans the new concept of a biological axis of iNKT cells and IL-33, involved in alerting and controlling the immune cells during sterile inflammation. However, our study is based on a single condition of sterile inflammation, namely LT, implying a population of patients with iNKT cell biology altered at baseline (see **Figure 1**, and (49, 50)) and having received immuno-suppression. In this clinical setting, we should therefore highlight the limitation of our phenotypic analysis, due to the very low events of iNKT cells, which are virtually absent in one third of patients.

Another limitation of our study is the origin of iNKT cells that are detected among recipient PBMC, namely transplant-derived versus host origin. Although microchimerism of hematopoietic cells has not been documented during transplantation procedure in LT recipients, we cannot exclude early recirculation of liver resident iNKT cells from the donor liver during this time-sequence. However, the fact that we did not observe an increase in the frequency of iNKT within PBMC in recipient patients between the beginning and the end of LT, suggests that this phenomenon, if it occurs, is still marginal.

An important objective now would be to determine whether the IL-33/iNKT cell biological axis contributes to the severity of IRI associated with LT. In fact, in our mouse model of liver IR, IL-33 and iNKT cells were separately deleterious as evidenced by attenuation of IRI in IL-33-deficient mice (40) and iNKT cell-deficient mice (see **Supplementary Figure 9** and **Supplementary Figure 10**), respectively. In a remarkably similar fashion, when considering the setting of human LT, we previously reported that IL-33 release at reperfusion was positively correlated with cardinal features of early liver injury-associated disorders after LT (40). The possibility that activation of iNKT cells is also closely associated with IR manifestations during LT, which is suggested in the present study by a trend toward positive correlation between the proportion of activated iNKT cells and the MEAF score, remains to be demonstrated in a larger cohort of patients.

Although the precise functions and associated mechanisms of iNKT cells necessitate further investigation, their possible action *via* IFN-γ or/and IL-17A secretion, as reported in mouse IRI models of kidney IRI (12, 13, 17), merits special attention.

Unlike the mouse, resident iNKT cells in human liver are not abundant (% among T lymphocytes: 0.1 to 1.0 *vs.* 5 to 40 in mouse) (51–53). Since our analysis is restricted to peripheral blood, it is difficult to determine whether activation of circulating iNKT cells mirrors their migration into the liver. However, this hypothesis is in line with our data in mice showing that iNKT cell activation in the periphery (spleen) driven by liver IR is accompanied by their recruitment to the liver. In all cases, we assume that both host-derived and graft-resided iNKT cells are targeted by IL-33, thereby causing graft dysfunction in the early phase of LT.

Another important aim of our study was to determine whether during LT, IL-33 also drives activation of the other two main circulating ITC components, namely MAIT and γδ-T cells. As expected, the two non-conventional T cell components shared with iNKT cells a preferential activation during LT, in accordance with their well-recognized hyperreactivity to endogenous cellular stress elements. Of note, as with iNKT cells, MAIT and γδ-T cells displayed a profound number defect in patients awaiting LT, in accordance with previous reports (49, 54), thereby suggesting a general and coordinated “innateness” alteration. Despite this, as was the case for iNKT cells, activation of MAIT cells during LT was closely associated with both early release of IL-33 and severity of liver dysfunction after graft reperfusion. Future studies using mice lacking MAIT cells (MR1-deficient mice) and IL-33-deficient mice overexpressing MAIT cells (IL-33^gt/gt^ x Vα19*i* TCR transgenic mice) will allow to directly test the existence of a deleterious IL-33/MAIT cell axis during liver IRI.

In clear contrast to activation of MAIT cells, activation of γδ-T cells during LT did not correlate with early release of IL-33. This difference may be explained by the fact that, unlike iNKT and MAIT cells (12, 38, 39, 46), γδ-T cells do not seem directly targeted by IL-33 (55). Of course, future studies dedicated to γδ-T cells during LT will need to take into account the significant qualitative differences between the two major γδ-T contingents present in the blood (i.e., δ1 and δ2).

Interestingly, this response pattern within the ITC compartment is similar to that reported in the acute response to COVID-19 infection (56), in which activation of all three ITC subsets also occurs. In this infection condition, activation of iNKT and MAIT cells, but not γδ-T cells, was correlated with both IL-18 secretion and disease severity. Since IL-18 is known to fully activate iNKT and MAIT cell functions in synergy with IL-33 (12, 38, 39, 46), it remains to be investigated whether IL-33 might be a common and key component in infection and sterile inflammation conditions, driving MAIT and iNKT cells to initiate the clinical course and to determine disease severity. In this context, special attention should also be given to the capacity attributed to iNKT and MAIT cells (57–60), as well as IL-33 (37), to lead to repair response. Longitudinal analysis after LT will help to determine whether MAIT and iNKT cell activation persists over time and may be associated with protective mechanisms in relation with IL-33.

More generally, additional analyses comparing other sterile inflammation conditions and infection of other origins will help to better evaluate the “sterile inflammation” specificity of our findings.

In summary, we have shown that LT drives general and preferential activation of the three main ITC components, namely iNKT, MAIT, and γδ-T cells. In addition, we have identified iNKT and MAIT cells in connection with IL-33 as new key cellular factors and mechanisms of sterile inflammation in clinical settings. Of importance, our study also raises the possibility that the levels of activation (as assessed by CD69 expression) on MAIT and iNKT cells may be predictive of the clinical course of sterile inflammation conditions. Further investigations will be required to precisely assess their functions during sterile inflammation and its resolution after LT.

Taken as a whole, these findings should encourage further studies on MAIT and iNKT cells in sterile inflammation conditions so as to assess their potential as biomarkers and/or targets for immune intervention strategies.

## Data availability statement

The raw data supporting the conclusions of this article will be made available by the authors, without undue reservation.

## Ethics statement

The studies involving human participants were reviewed and approved by Comité consultatif de protection des personnes dans la recherche biomédicale Tours-Région Centre-Ouest 1 under registration number DC-2016-2651. The patients/participants provided their written informed consent to participate in this study. The animal study was reviewed and approved by COMETHEA Poitou Charentes, authorization no. 2016110211568800.

## Author contributions

AR and CM developed methodology, performed investigation, and wrote the original draft. RB performed investigations and reviewed the manuscript. AB performed formal analysis and reviewed the manuscript. FD helped in creating the liver injury score and in analyzing liver tissue. AT, TH, and LP were involved in conceptualization and project administration and reviewed the manuscript. J-MG was involved in conceptualization, performed supervision and reviewed the manuscript. ES and AH were involved in conceptualization and project administration, performed supervision, and reviewed the manuscript. LB developed methodology, performed investigations, was involved in conceptualization and project administration, performed supervision, and wrote the original draft. All authors contributed to the article and approved the submitted version.
