# Supplementary material for: Early activation and recruitment of invariant natural killer T cells during liver ischemia-reperfusion: the major role of the alarmin interleukin-33
Source: Front Immunol. 2023 May 9;14:1099529. doi: 10.3389/fimmu.2023.1099529 (PMC10203422; doi:10.3389/fimmu.2023.1099529)
Supplement: Supplementary file 1 [file Table_1.pdf]

Recipient characteristics	n=21
Recipient age (years)	55 (±2.2)
MELD score	18.2 (±2.4)
Indication for LT :	
Hepatocellular carcinoma	1 (5%)
Alcoholic liver disease	14 (67%)
Hepatitis C	3 (14%)
Other	3 (14%)
**Donor and LT characteristics**	
Donor age (years)	50.2 (±4.5)
Extended criteria grafts*	10 (48%)
Cold Ischemia time (min)	370 (±24)
Temporary portocaval shunt	10 (48%)
**Outcomes**	
Ischemia-reperfusion syndrome	8 (38%)
MEAF score	5.2 (±0.3)
Primary non function	0 (0%)
Clavien-Dindo ≥ 3	0 (0%)
Length of stay (days)	8 (±1)
Acute kidney injury requiring dialysis	1 (5%)
1-y graft survival rate	21 (100%)

Supplementary Table 1: Baseline demographic, related outcome measures, and clinical information of the study group of LT patients (n=21). Qualitative variables are expressed as absolute numbers and %. Quantitative variables are expressed as (mean±SEM). MELD, model for end-stage liver disease; MEAF, Model for early allograft function. *Extended criteria for liver grafts according to Eurotransplant definition : donor age >65 years, intensive care unit stay with ventilation >7 days, body mass index >30 kg/m2, steatotic liver >40%, serum sodium >165 mmol/L, AST >105 UI/L, ALT >90 UI/L, serum bilirubin >3 mg/dL, donation after circulatory death.
