# Supplementary material for: Early activation and recruitment of invariant natural killer T cells during liver ischemia-reperfusion: the major role of the alarmin interleukin-33
Source: Front Immunol. 2023 May 9;14:1099529. doi: 10.3389/fimmu.2023.1099529 (PMC10203422; doi:10.3389/fimmu.2023.1099529)
Supplement: Supplementary file 2 [file Table_2.pdf]

Antibodies/Reagents	*Conjugate*	*Specificity*	*Provider*	*Clone*
Anti-pan TCRγδ	BUV661	Human	BD biosciences	11f2
Anti-TCR Vα24-Jα18	BUV805	Human	BD biosciences	6B11
5-OP-RU MR1 tetramer	APC	Human	NIH	
Anti-CD161	BV480	Human	BD biosciences	HP-3G10
Anti-CD69	BV750	Human	BioLegend	FN50
Anti-CD3	Spark550	Human	BioLegend	SK7
Anti CD4	cFluor YG584	Human	Cytek	SK3
Anti CD8	APC-Fire810	Human	BioLegend	SK1
Live/dead	blue	Human	Thermo Fischer	
Zombie NIR fixable kit	APC-Cy7	Mouse	BioLegend	
Anti-CD45	Alexa Fluor 488	Mouse	BioLegend	30-F11
Anti-TCRαβ	PerCpCy5.5	Mouse	BioLegend	H57
PBS57 CD1d tetramer	PE	Mouse	NIH tetramer Core Facility	
Anti-CD69	BV421	Mouse	BD biosciences	H1.2F3
Anti-CDllb	PE	Mouse	BioLegend	M1/70
Anti-GR-1	BV421	Mouse	BioLegend	RB6-8C5

Supplementary Table 2. List of conjugated antibodies and reagents for flow cytometry in human and mouse immune cells.
