# Supplementary figures and images for: Early activation and recruitment of invariant natural killer T cells during liver ischemia-reperfusion: the major role of the alarmin interleukin-33
Source: Front Immunol. 2023 May 9;14:1099529. doi: 10.3389/fimmu.2023.1099529 (PMC10203422; doi:10.3389/fimmu.2023.1099529)

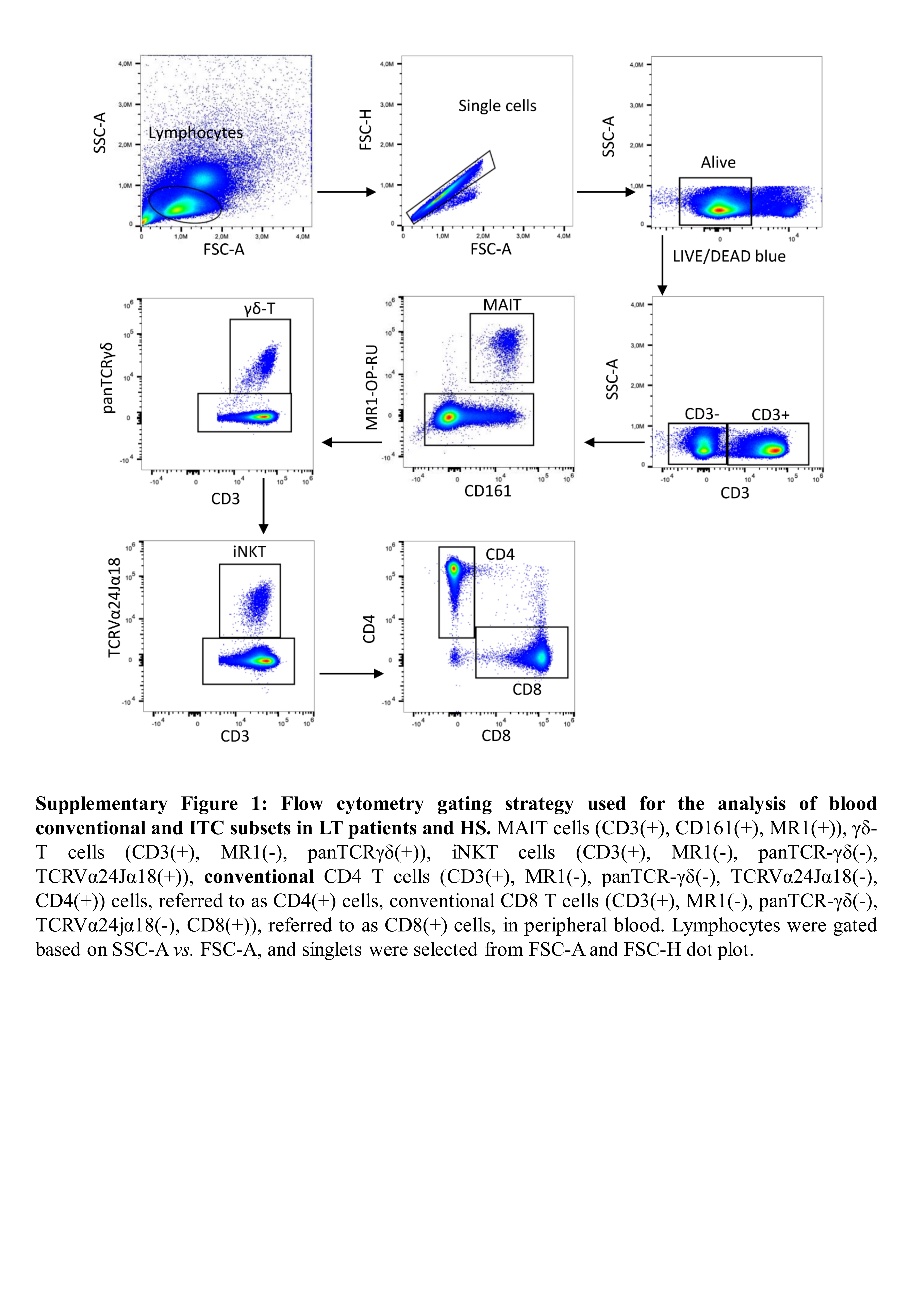

Supplement: Supplementary file 3 [file Image_1.jpg]

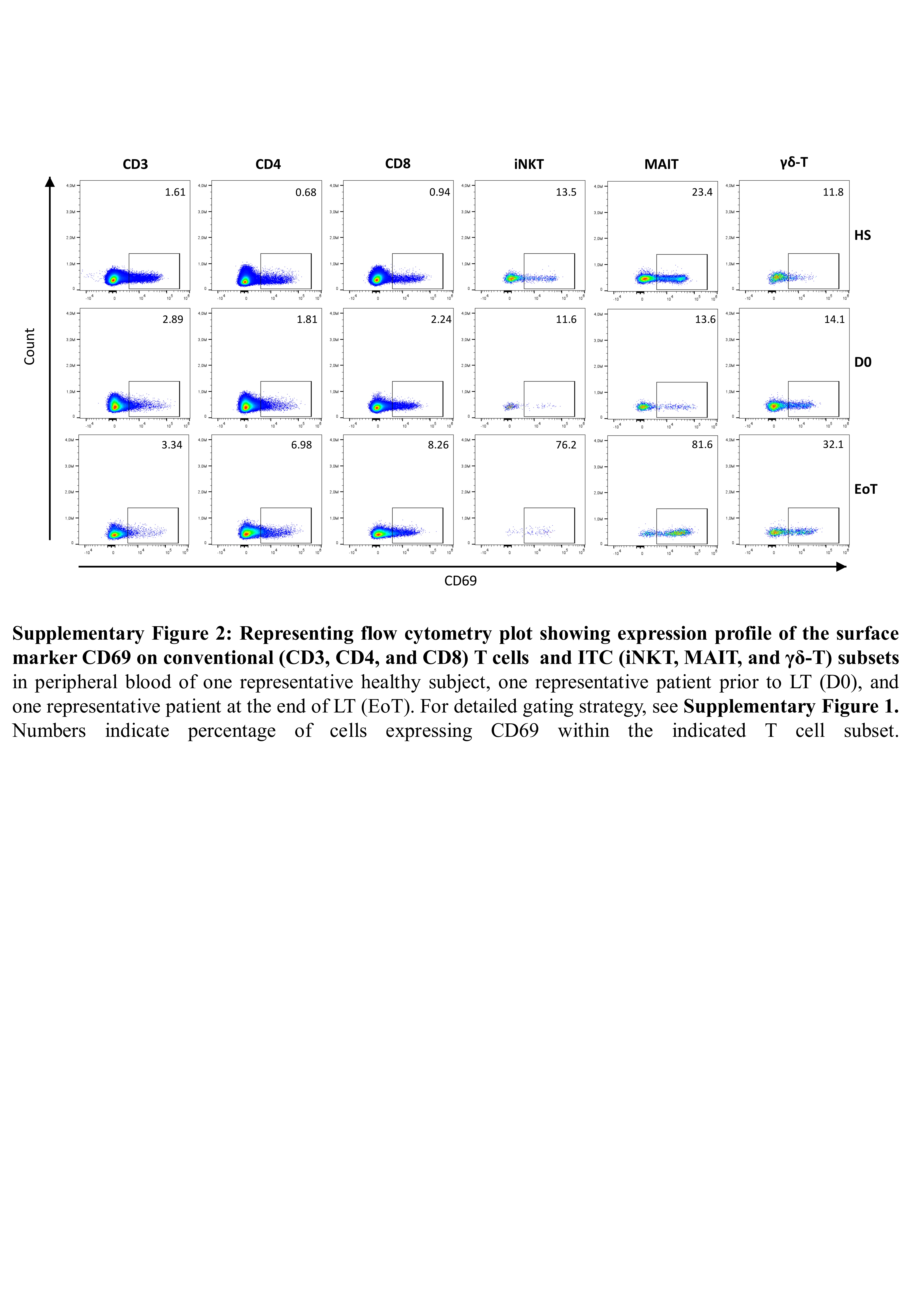

Supplement: Supplementary file 4 [file Image_2.jpg]

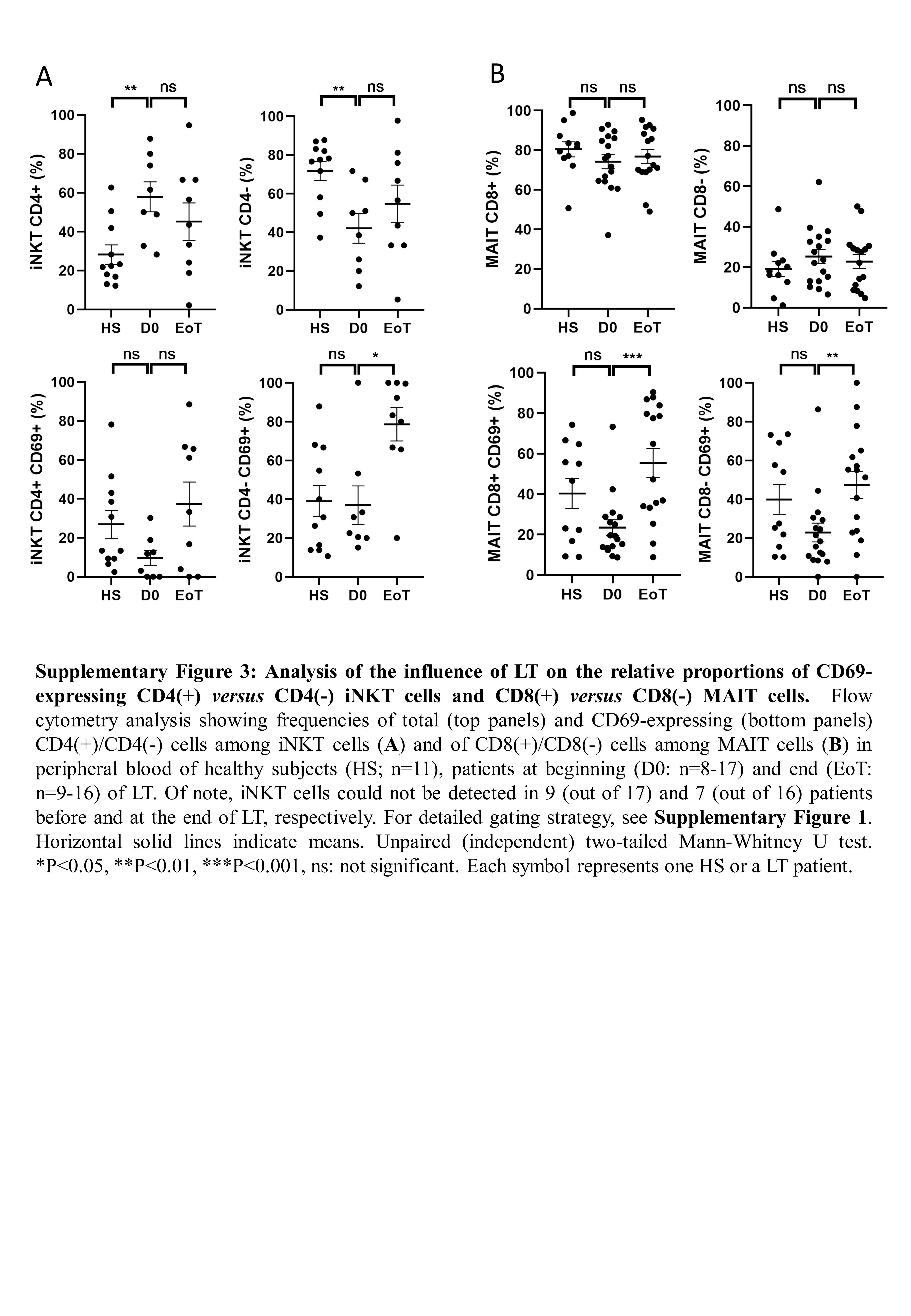

Supplement: Supplementary file 5 [file Image_3.jpg]

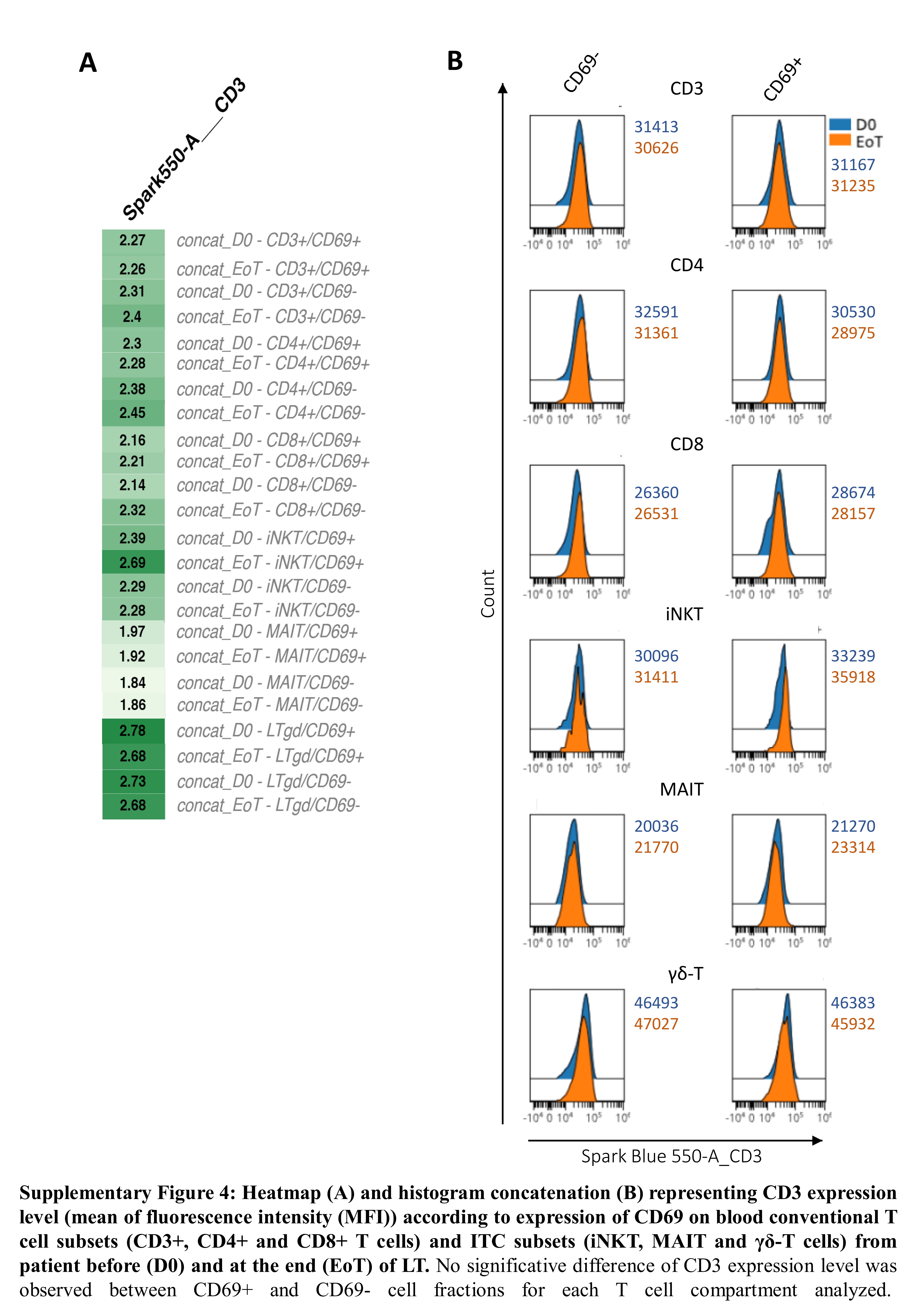

Supplement: Supplementary file 6 [file Image_4.jpg]

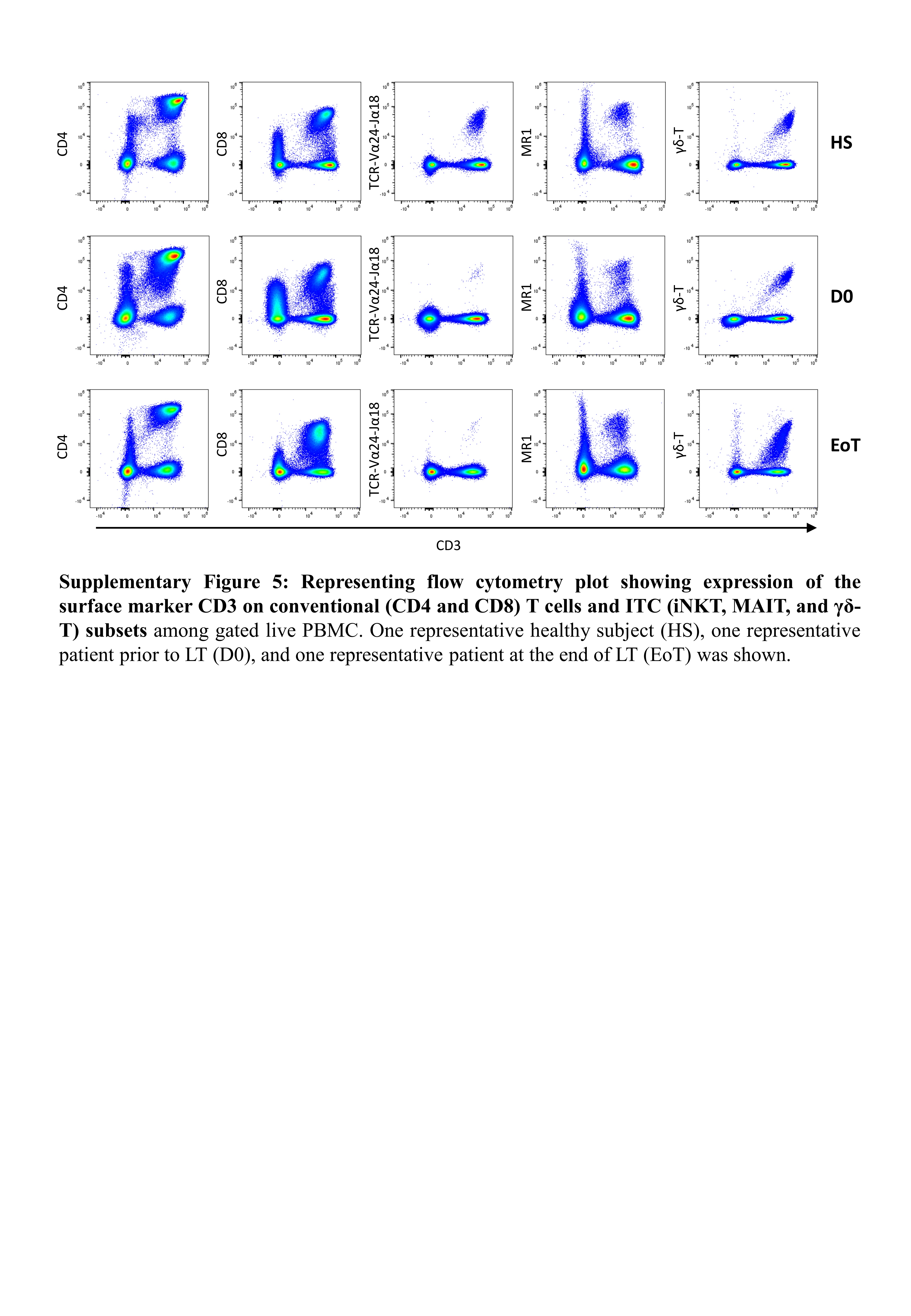

Supplement: Supplementary file 7 [file Image_5.jpg]

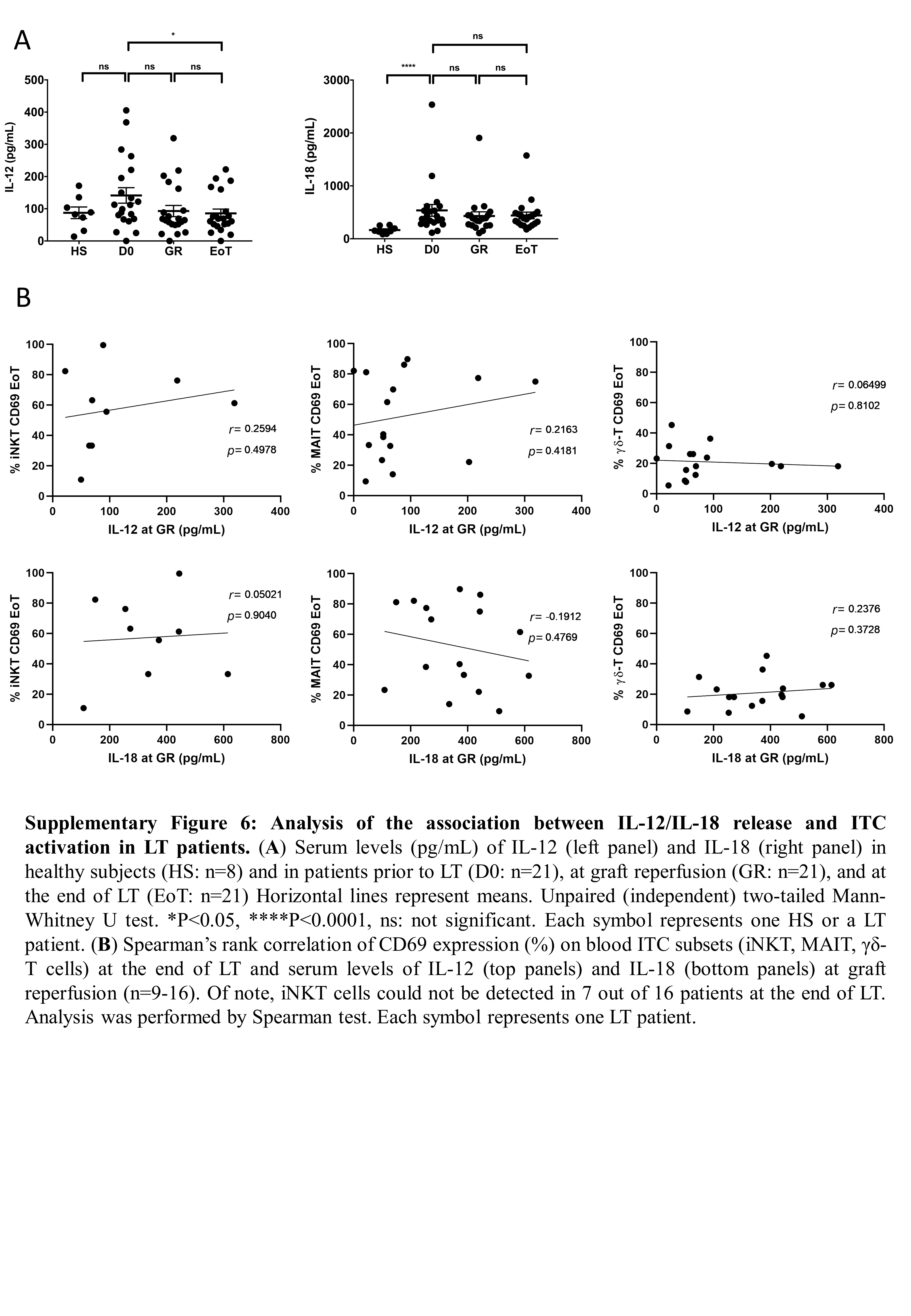

Supplement: Supplementary file 8 [file Image_6.jpg]

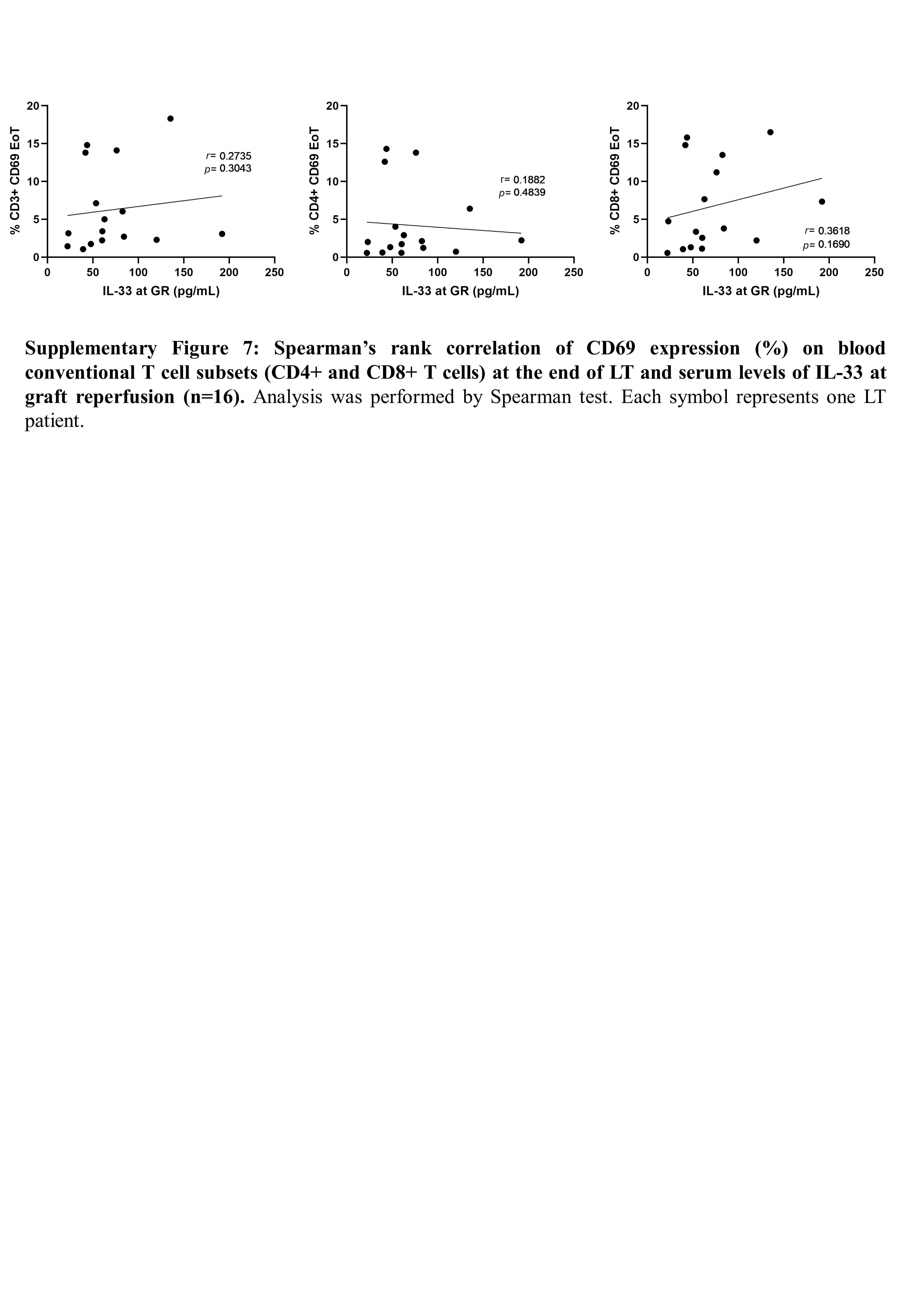

Supplement: Supplementary file 9 [file Image_7.jpg]

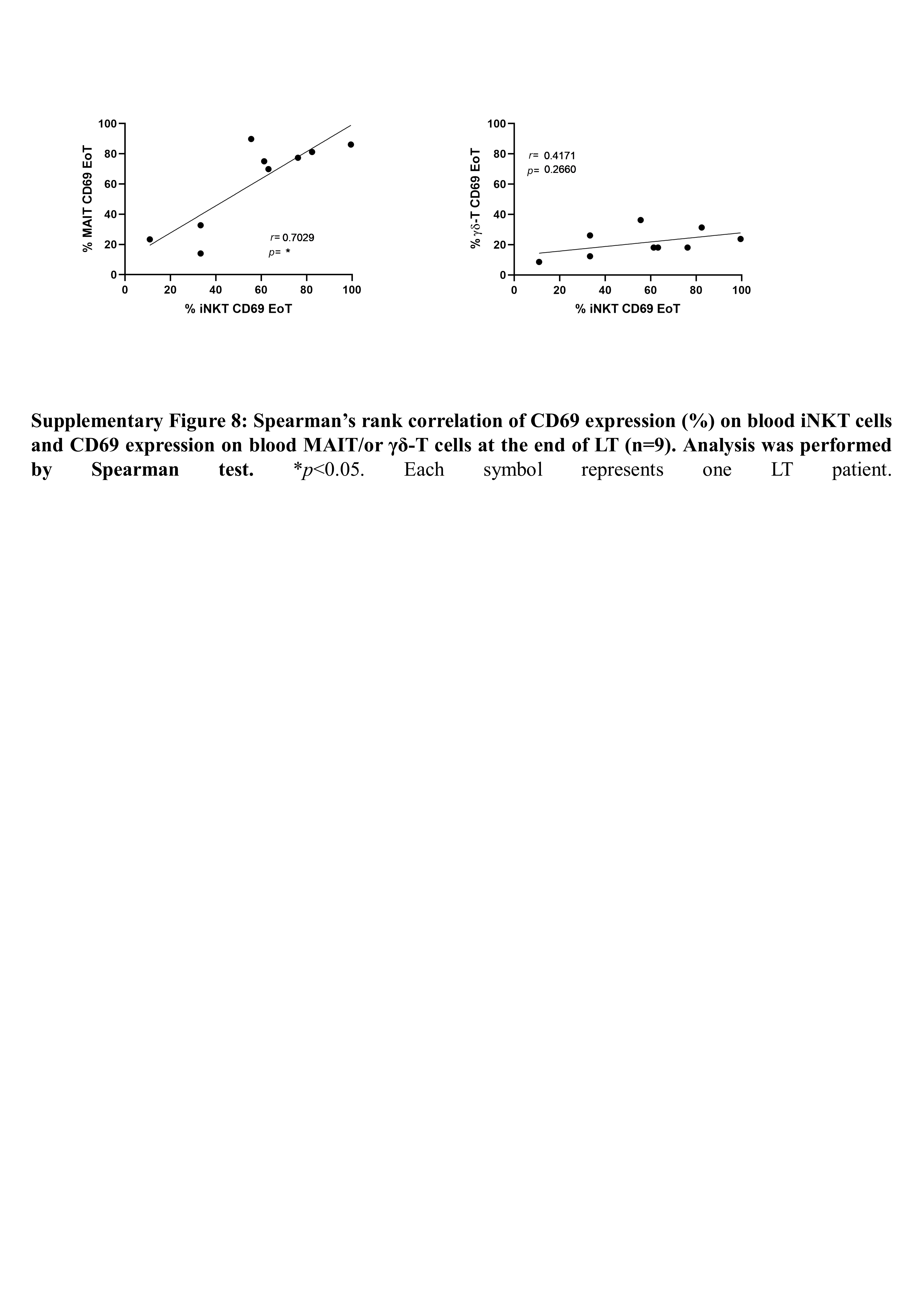

Supplement: Supplementary file 10 [file Image_8.jpg]

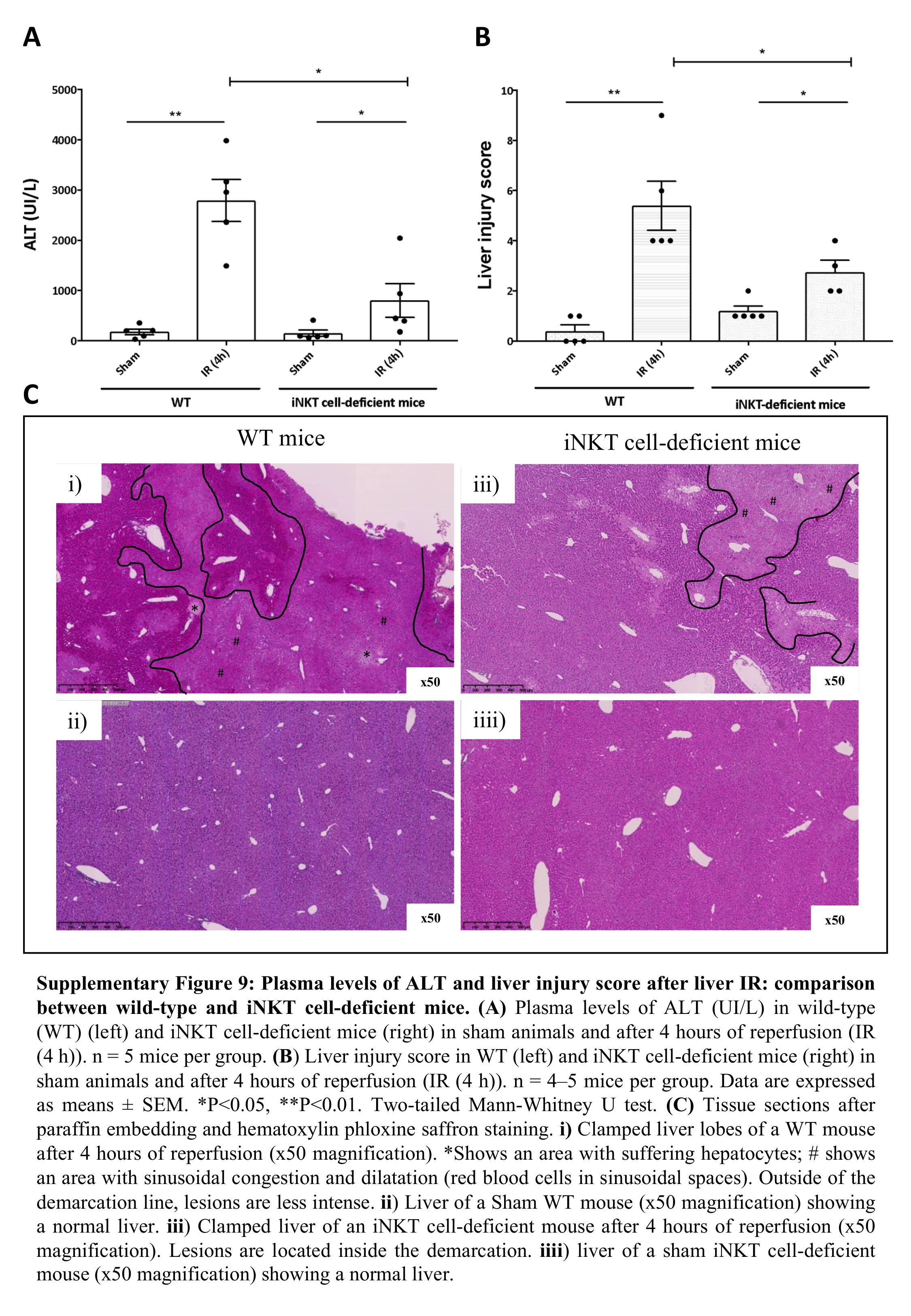

Supplement: Supplementary file 11 [file Image_9.jpg]

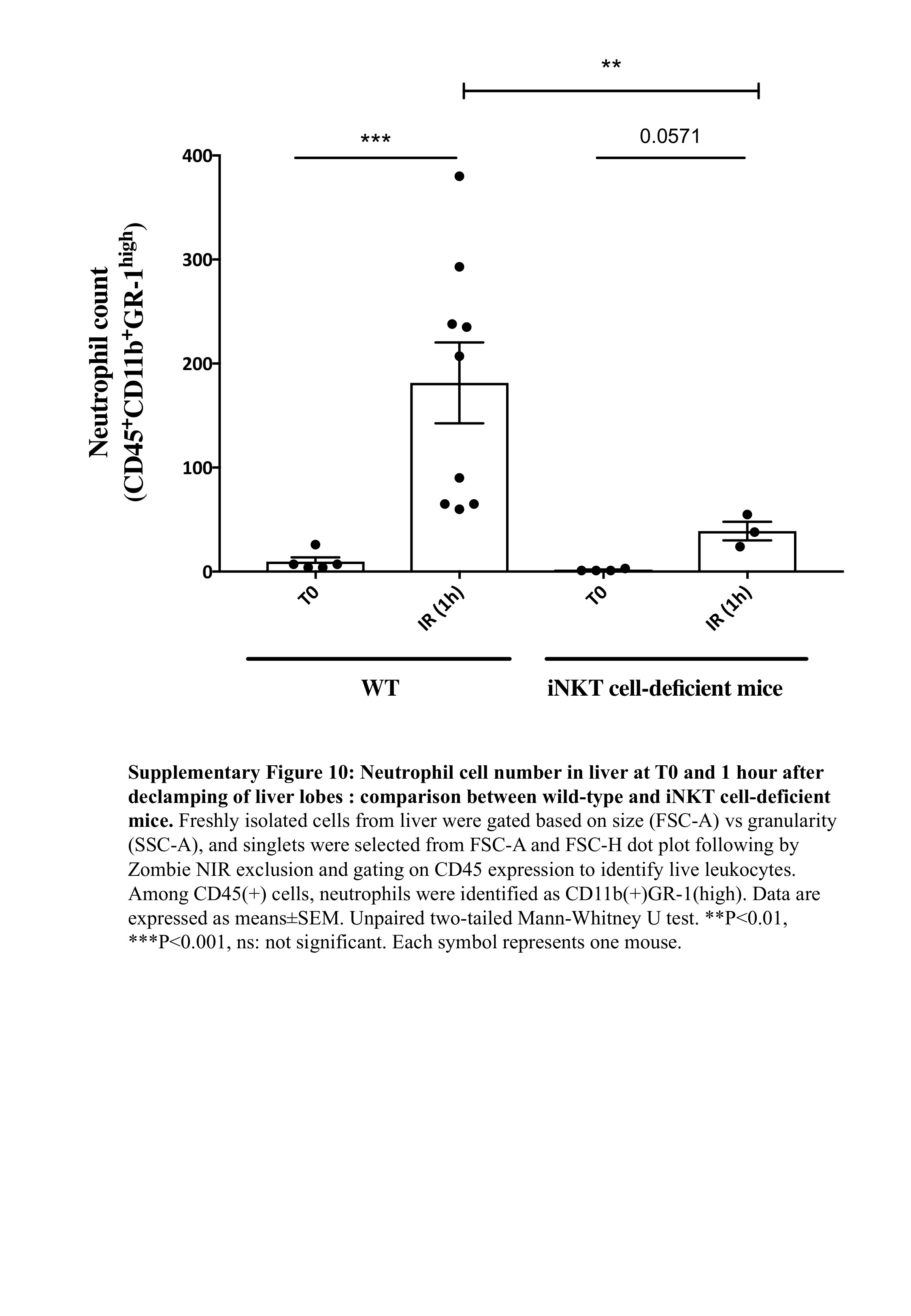

Supplement: Supplementary file 12 [file Image_10.jpg]

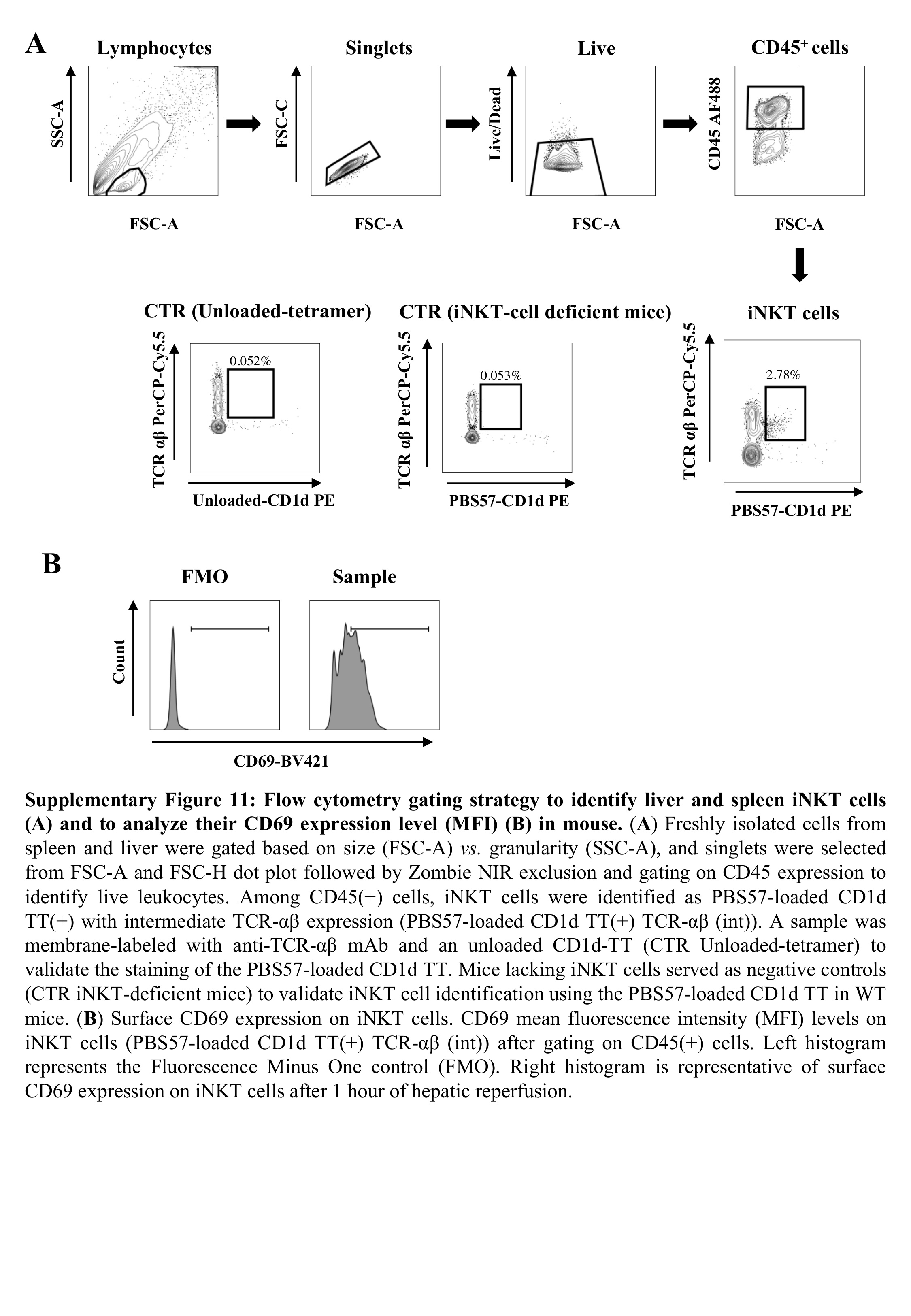

Supplement: Supplementary file 13 [file Image_11.jpg]

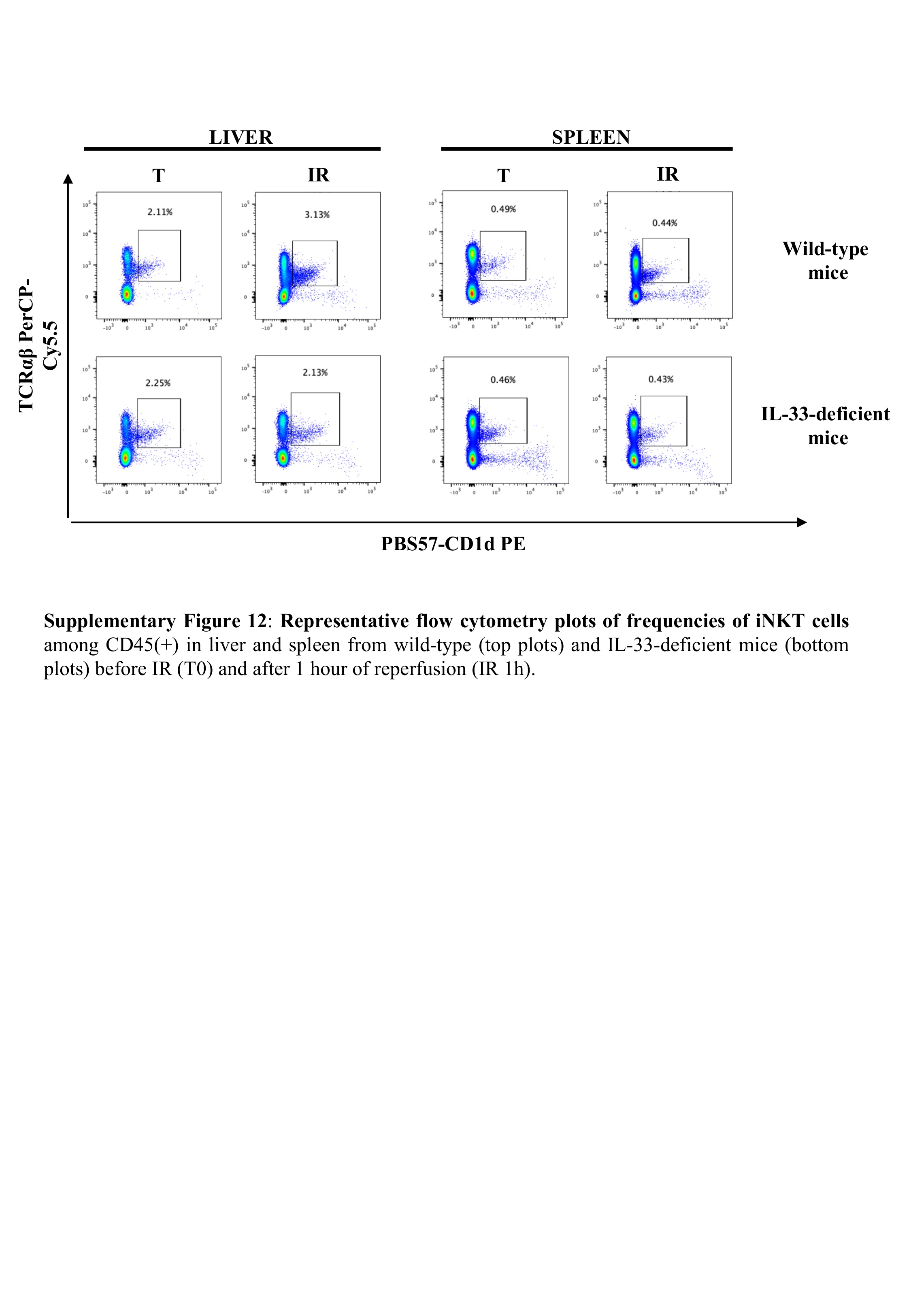

Supplement: Supplementary file 14 [file Image_12.jpg]

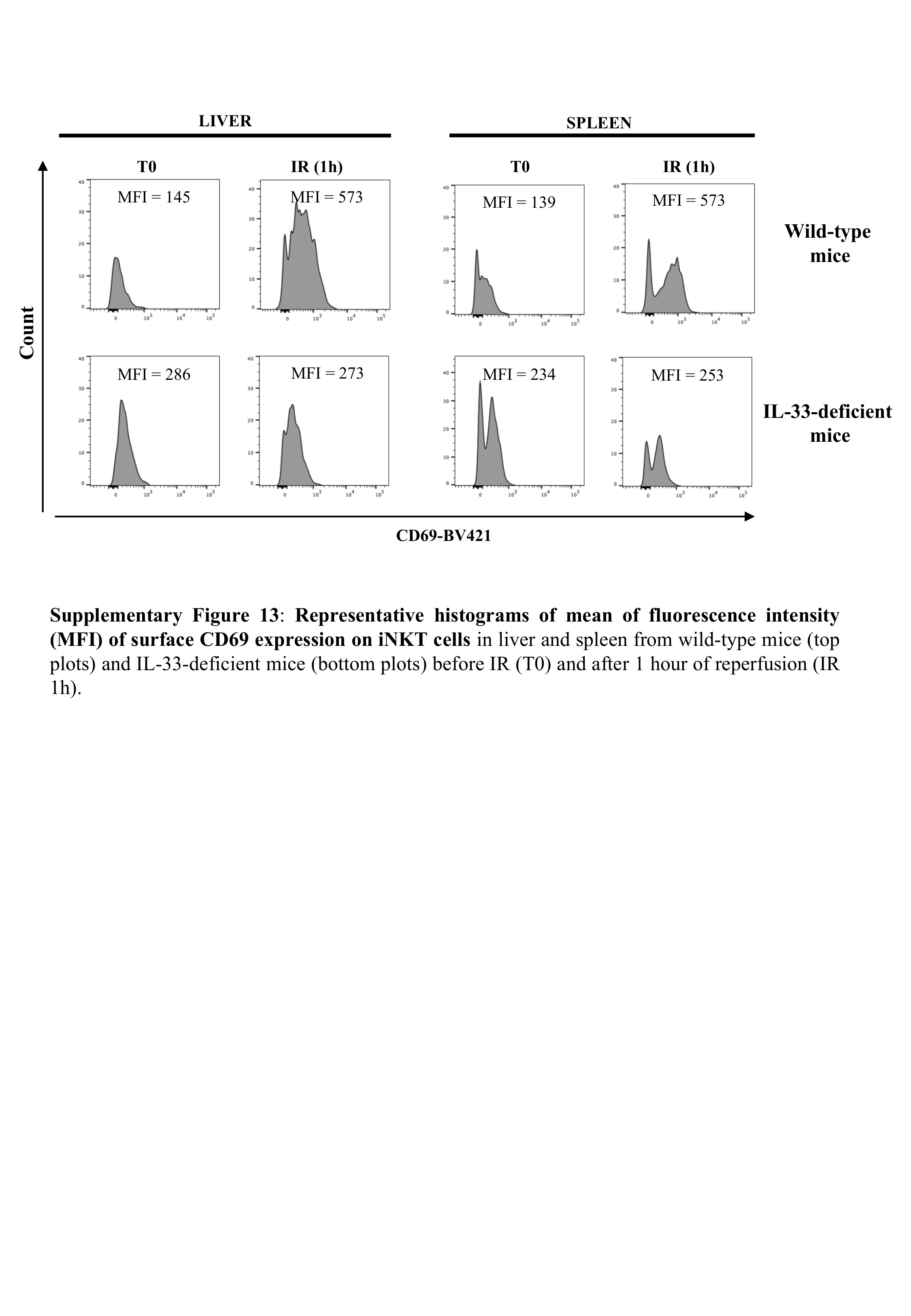

Supplement: Supplementary file 15 [file Image_13.jpg]
